# Fast and Sensitive Detection of Pb^2+^ in Foods Using Disposable Screen-Printed Electrode Modified by Reduced Graphene Oxide

**DOI:** 10.3390/s131013063

**Published:** 2013-09-26

**Authors:** Jin-Ming Jian, Yan-Yan Liu, Ye-Lei Zhang, Xi-Shan Guo, Qiang Cai

**Affiliations:** 1 College of Biosystems Engineering and Food Science, Zhejiang University, Hangzhou 310058, China; E-Mails: jianjinming@zju.edu.cn (J.-M.J.); lyy3192@126.com (Y.-Y.L.); 2 Linyi University, Linyi 276005, China; E-Mail: cherrylanlan@126.com; 3 Yangtze Delta Region Institute of Tsinghua University, Jiaxing 314006, China; E-Mail: caiq@tsinghua.edu.cn

**Keywords:** reduced graphene oxide (rGO), electrochemical deposition, screen-printed carbon electrode (SPCE), square wave voltammetry, Pb^2+^

## Abstract

In this study, reduced graphene oxide (rGO) was electrochemically deposited on the surface of screen-printed carbon electrodes (SPCE) to prepare a disposable sensor for fast detection of Pb^2+^ in foods. The SEM images showed that the rGO was homogeneously deposited onto the electrode surface with a wrinkled nanostructure, which provided 2D bridges for electron transport and a larger active area for Pb^2+^ adsorption. Results showed that rGO modification enhanced the activity of the electrode surface, and significantly improved the electrochemical properties of SPCE. The rGO modified SPCE (rGO-SPCE) was applied to detect Pb^2+^ in standard aqueous solution, showing a sharp stripping peak and a relatively constant peak potential in square wave anodic stripping voltammetry (SWASV). The linear range for Pb^2+^ detection was 5∼200 ppb (R^2^ = 0.9923) with a low detection limit of 1 ppb (S/N = 3). The interference of Cd^2+^ and Cu^2+^ at low concentrations was effectively avoided. Finally, the rGO-SPCE was used for determination of lead in real tap water, juice, preserved eggs and tea samples. Compared with results from graphite furnace atomic absorption spectroscopy (GFAAS), the results based on rGO-SPCE were both accurate and reliable, suggesting that the disposable sensor has great potential in application for fast, sensitive and low-cost detection of Pb^2+^ in foods.

## 1. Introduction

Recently, heavy metal contamination in food sources has become a very serious problem, especially in the developing countries. The most terrible consequence is that heavy metals cannot be eliminated from foodstuffs by soaking, washing, cooking or other methods. According to the stipulation of the World Health Organization (WHO), levels of lead, cadmium, chromium, and other heavy metals must definitely be controlled in food sources in order to assure the public safety [1]. The US Environmental Protection Agency (EPA) has determined that lead is a probable human carcinogen, which can damage every organ and system in human body. Exposure to lead (Pb) at high levels can severely damage the brain and kidneys, and even cause death to human beings. For pregnant women, high levels of exposure to lead may cause miscarriages [2], so it is critical to control and determine the heavy metal concentrations in food.

Due to the demand for monitoring heavy metal ions, a series of techniques have been developed in the past, including atomic absorption spectrometry [3–6], inductively coupled plasma mass spectrometry [7], inductively coupled plasma atomic emission spectroscopy [8] and X-ray fluorescence spectrometry [9]. Conventional methods can detect heavy metal sensitively and accurately, but have drawbacks like complicated sample pre-treatment, time-consuming process, expensive cost, large-scale analysis for indoor use, and requirement of expert operators. In recent years, electrochemical sensors and analysis methods have been developed for heavy metal detection, due to their advantages of small size, high sensitivity, low cost and easy operation. Ghanjaoui applied a system based on bismuth film electrodes combined with a rotating disk electrode to determine lead in beer samples, which displayed excellent linear behavior to 5 to 85 μg/L (5∼85 ppb) lead with a detection limit of 0.27 μg/L (0.27 ppb) [10]. Ping used an ionic liquid and a bismuth oxide nanoparticle-modified carbon paste electrode to determine trace metals in milk, and the linear detection range for Pb^2+^ was from 3.0 to 30.0 μg/L (3∼30 ppb) with a detection limit of 0.21 μg/L (0.21 ppb) [11]. The aforementioned electro-analytical methods, especially stripping analysis, have been identified as the most promising approaches to meet this analytical need [12]. However, most solid electrodes have some drawbacks, such as cross-contamination, electrode surface poisoning and other issues that may affect the analysis results, while the disposable screen-printed carbon electrode (SPCE) can efficiently overcome these problems. Moreover, the SPCE shows a heterogeneous nature that is similar to that of carbon paste electrodes. Recently, a novel hanging galinstan drop electrode (HGDE) based on a composite of liquid metal marbles and WO_3_ nanoparticles has been developed [13]. The as-prepared HGDE with simple surface renewal and predictable electrochemical properties, could sensitively and selectively determine Pb^2+^ and Cd^2+^ at low concentrations, but it still has some limitations for on-site applications, as compared with the SPCE which is characterized by low cost, easy miniaturization and great potential for mass production.

A screen-printed electrode combined with anodic stripping voltammetry (ASV) is a very promising method for heavy metal detection. Keawkim determined lead in rice by sequential injection/anodic stripping voltammetry using a bismuth/crown ether/Nafion film-modified SPCE. They found a linear detection range of 0.5 to 60 mg/L (0.5∼60 ppm) for Pb^2+^, with a detection limit of 0.11 mg/L (110 ppb) [14]. Kadara reported a disposable bismuth oxide-modified SPCE for detection of lead in water samples, which displayed excellent linear behavior in the concentration range of 20 to 300 μg/L (20∼300 ppb) with a detection limit of 8 μg/L (8 ppb) [15]. Mandil reported SPCE modified by gold films, which displayed excellent linear behavior in the Pb^2+^ concentration range from 2 to 16 μg/L (2∼16 ppb) with a detection limit of 0.5 μg/L (0.5 ppb) [12]. Injang developed screen-printed carbon nanotube electrodes with sequential injection analysis-anodic stripping voltammetry. The result showed that the linear range was 2∼100 μg/L (2∼100 ppb) for Pb^2+^, and the limit of detection was 0.2 μg/L (0.2 ppb) [16].

This study aims to extend the linear range and improve the sensitivity based on a method combining SPCE with stripping voltammetry, which has the advantages of being disposable, convenient and accurate. Due to the excellent electrical, mechanical, and thermal properties, graphene attracts popular attention as a new 2D nanomaterial, which can significantly improve the electrochemical properties, including sensitivity, selectivity and stability of screen-printed electrodes modified by graphene derivatives [17,18]. In this study, a disposable sensor based on rGO-SPCE was developed for fast, sensitive and low-cost determination of lead in food samples. The rGO was electrochemically deposited on the surface of bare SPCE to improve its electrochemical properties. The rGO-SPCE was applied in Pb^2+^ detection combined with square wave voltammetry. The modification process, characterization, detection performance, interference tests and real samples detection are explored.

## 2. Experimental Section

### 2.1. Material and Apparatus

Graphene oxide (GO) was purchased from Nanjing XF Nano Materials Tech Co. Ltd, Nanjing, China. The bare screen-printed electrodes (SPEs) were fabricated with a DEK248 printer machine (DEK, Weymouth, UK). The disposable sensor consists of a miniaturized three-electrode system including an Ag/AgCl pseudo-reference electrode, and two carbon electrodes acting as working and counter electrode, respectively. The diameter of the working electrode was 3 mm. K_3_[Fe(CN)_6_], KCl, Pb(NO_3_)_2_, Na_2_HPO_4_, NaH_2_PO_4_, acetic acid (HAc) and all other chemicals used were of analytical grade. Deionized water (18.2 MΩ·cm at 25 °C, Milli-Q) was used throughout the experiments. Tap water was obtained from the laboratory. Orange and apple juice, green and black tea bags (1.5 g tea powder per bag, produced in Anhui Province, China), and three kinds of preserved eggs were purchased from a local supermarket. The electrochemical measurements were achieved by Autolab PGSTAT128N (Metrohm, Herisau, Switzerland) at ambient temperature (25 °C). The morphology of modified working electrode on the SPCE was observed by a field emission scanning electron microscope (Hitachi SU70, Tokyo, Japan). Raman spectra were recorded using a LabRAM HR UV800 Raman Spectroscopy (Jobin, Paris, France) with a 632 nm HeNe laser.

### 2.2. Preparation of the rGO-SPCE

**Figure 1.**
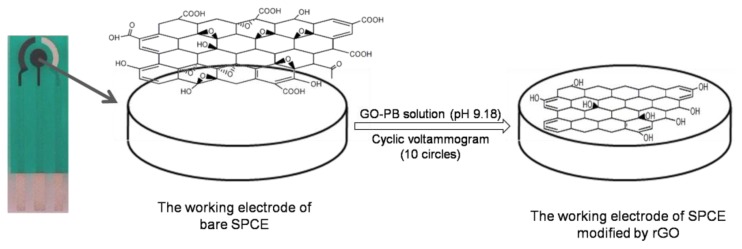
Schematic of electrochemical deposition of rGO on the surface of working electrode in the SPE.

Graphene oxide powder was dispersed in 0.067 M phosphate buffer (PB, pH 9.18) and ultrasonicated for 2 h to obtain a uniform suspension containing 1 mg/mL GO. Then the GO suspension (80 μL) was dropped on the surface of the SPE, and then scanned from −1.4 V to 0 V for 10 cycles with a scan rate of 50 mV/s. As a result, the reduced GO was electrochemically deposited onto the working electrode surface via cyclic voltammetry (CV) [19,20]. The modified rGO-SPCE was washed carefully with double distilled water, and dried in air. Figure 1 shows a schematic of the electrochemical deposition as well as the process of GO reduction which occurred on the SPCE surface. The determination of electrochemical properties of the SPCE before and after modification of rGO was performed in 5 mM K_3_[Fe(CN)_6_]/0.1 M KCl solution. The morphologies of the bare and modified working electrode were observed by the field emission scanning electron microscope (FE-SEM).

### 2.3. Pre-Treatments of Real Samples

Tap water, apple juice, orange juice, preserved eggs, black tea and green tea were selected as real food samples. Different samples were pre-treated by different fast pretreatment process for on-site real-time field measurement of Pb^2+^ in foods. Tap water was filtered through a 0.22 μm membrane (Millipore, Billerica, MA, USA), and the pH was adjusted to 4.2∼4.5 using 1 M NaOH/HAc solution.

For fruit juice, 10 mL of sample was added to 10 mL of 0.1 M HAc solution. After shaking for 20 min, the mixture was filtered and the pH was adjusted to be 4.2∼4.5 with NaOH/HAc solution.

Standard addition method was applied for tap water, orange juice and apple juice. Briefly, 5 mL of Pb^2+^ solution, of which the Pb^2+^ concentration was respectively 10 ppb, 30 ppb and 90 ppb, was fully mixed with 5 mL of the pre-treated sample solution.

For black tea and green tea, 1.0 g of tea powders was added to 25 mL of 0.1 M HAc solution and allowed to stand for 30 min. Then the mixture was filtered and the pH was adjusted to 4.2∼4.5 by NaOH/HAc solution.

For preserved eggs, 10.0 g of the sample was added to 100 mL of 0.1 M HAc solution. After ultrasonication for 30 min, the mixture was filtered and the pH was adjusted to 4.2∼4.5 by NaOH solution.

### 2.4. Procedure for the SWASV Analysis

Firstly, the pre-treated sample solution was mixed with 0.1 M acetate buffer solution (pH 4.5) at the volume ratio of 1:2. Then 80 μL of the mixture was dropped onto the rGO-SPCE. The heavy metal detection was performed by square wave anodic stripping voltammetry (SWASV) with optimized parameters. The accumulation potential (−1.2 V) was applied to the rGO-SPCE for 420 s. After the pre-concentration step, the voltammograms were recorded by applying a positive-going square-wave potential scan (with amplitude of 30 mV, step potential of 2 mV, frequency of 20 Hz). The scanning was terminated at 0.3 V.

## 3. Results and Discussion

### 3.1. Characterization of the rGO-SPCE

**Figure 2.**
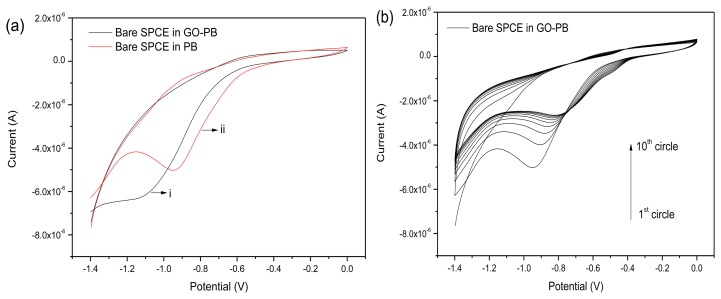
(**a**) CVs of bare SPCE in phosphate buffer (i) and phosphate buffer containing GO (ii) for the first circle; (**b**) CVs of bare SPCE in phosphate buffer containing GO (10 cycles).

The rGO was electrochemically deposited on SPCE via cyclic voltammetry in the GO suspension. Figure 2 shows the cyclic voltammograms of the bare SPCE in phosphate solution (Figure 2a) and GO suspension (Figure 2b).

As shown in Figure 2a, a cathodic peak at about −0.9 V appeared in the CV curve of the bare SPCE in the GO suspension, which should be attributed to the irreversible chemical reduction of several oxygen-containing functional groups on the exfoliated GO sheets. As the scan cycle increased, the cathodic peak gradually and positively shifted and the peak current decreased (Figure 2b). This result may be related to the deposited layer of GO sheets and the degree of reduction of the GO on the SPCE surface.

**Figure 3.**
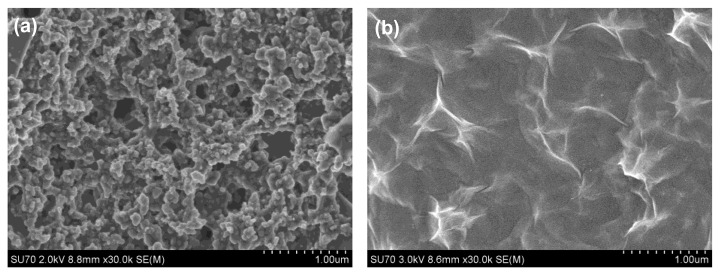
SEM images of (**a**) bare SPCE and (**b**) rGO modified SPCE as working electrode.

The SEM morphologies of the bare SPCE and rGO-SPCE are respectively shown in Figure 3. From Figure 3a, it can be seen that there were some impurities on the surface of bare SPCE. Based on Figure 3b, it was obvious that the rGO film was homogeneously deposited onto the electrode with a typical crumpled and wrinkled sheet structure. The wrinkled nanostructure of rGO sheets could provide a larger active area for the deposition of heavy metal ions in the preconcentration step.

**Figure 4.**
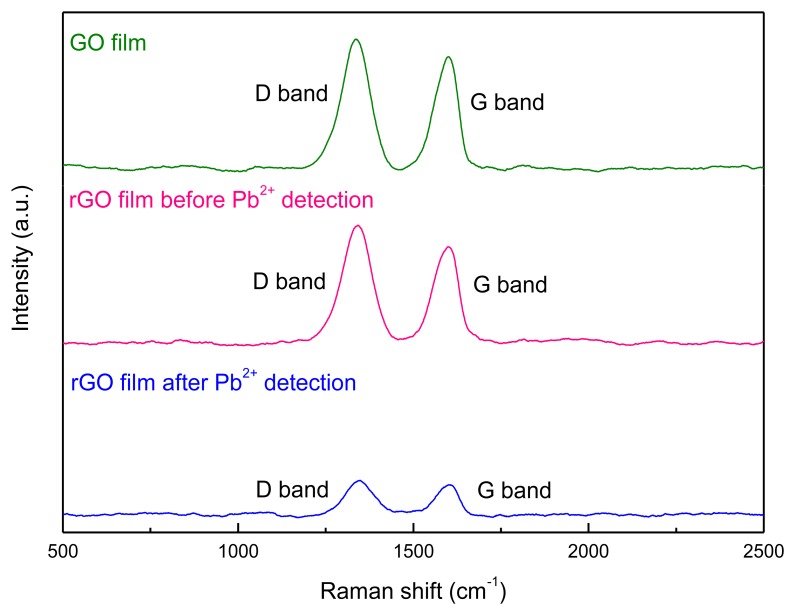
The Raman spectrum of GO film, rGO films before and after Pb^2+^ detection.

Raman spectroscopy was employed to explore the structural changes of GO caused by the electrochemical reduction. The Raman spectrum of GO sheets is characterized by two main components, the D band (1,330∼1,360 cm^−1^) originating from the edges which can be attributed to either defects or to the breakdown of translational symmetry [21], and the G band (1,580∼1,600 cm^−1^) corresponding to the first order scattering of the E_2g_ phonon of sp^2^ carbon atoms (Figure 4). For the produced rGO after electrochemical reduction, the intensity ratio (I_D_/I_G_) of D band to G band increased (Table 1). It could be deduced that the reduction had induced a decrease in the size of in-plane sp2 domains and an expansion of the disorder in the rGO [22]. The structural changes of rGO film after the detection of Pb^2+^ were also studied (Figure 4). It could be found that I_D_/I_G_ decreased slightly. This should be possibly caused by the residual Pb on the rGO film after stripping procedure, which occupy and thus reduce the defects on the edges of rGO film.

**Figure 5.**
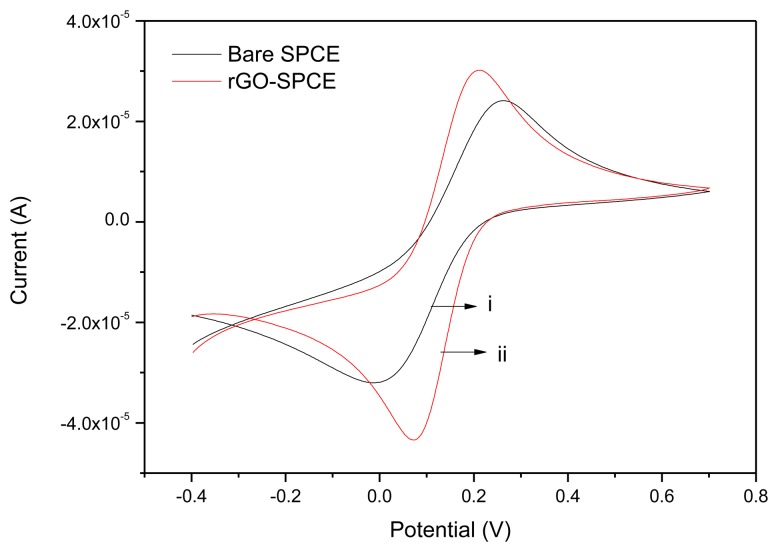
CVs of bare SPCE(i) and rGO-SPCE(ii) in 5 mM K_3_[Fe(CN)_6_]/0.1 M KCl solution. Scan rate: 50 mV/s.

In order to explore the variation in the surface chemistry of the SPCE before and after modification of rGO, the electrochemical properties of the SPCE were evaluated using the Fe(CN)_6_^3−/4−^ redox probe (Figure 5). The peak current and peak-to-peak potential separation both act as important indicators to reflect the charge transfer properties at the electrode surface. As shown in Figure 5, a pair of well-defined and quasi-reversible redox peaks was observed at the CVs both of bare SPCE and rGO-SPCE. The anodic peak current of rGO-SPCE (*ca.* 3.91 × 10^−5^ A, line ii) is larger than that of the bare SPCE (*ca.* 2.88 × 10^−5^ A, line i), indicating the increased active area of the SPCE after deposition of rGO sheets. The peak-peak potential separation of the rGO-SPCE is about 140 mV (line ii) while that of the bare SPCE is 260 mV (line i), suggesting the faster electron transfer at the rGO-SPCE surface [23] Therefore, the electrochemical deposition of rGO increased the active sites of the electrode surface, promoted the electron transfer, and thus improved electrochemical properties of the SPCE.

### 3.2. Detection of Pb^2+^ Using rGO-SPCE

The accumulation potential and accumulation time are the most critical factors for SWASV analysis. Here, the deposition potential and deposition time were respectively optimized by testing of the standard solution containing 50 ppb Pb^2+^.

#### 3.2.1. Optimization of Accumulation Potential

**Figure 6.**
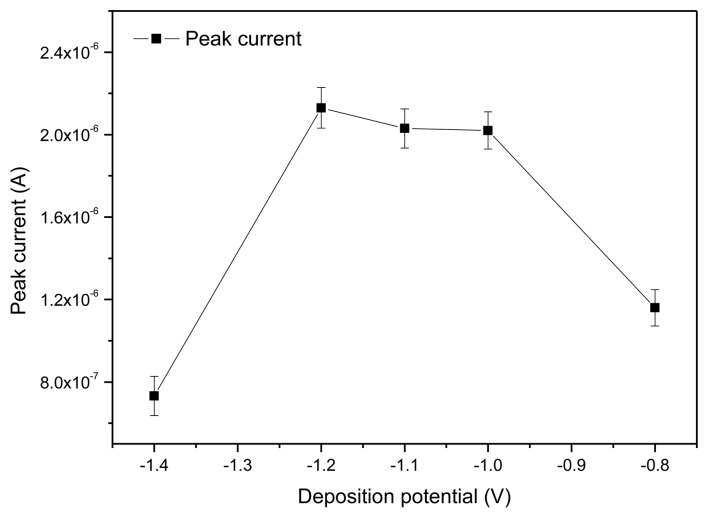
Effect of deposition potential on the peak current response. The data for respective error bar were obtained from four separate experiments.

As shown in Figure 6, the peak current gradually increased when the deposition potential increased from −1.4 V. When the deposition potential was up to −1.2 V, the maximum peak current was obtained. Then after a relatively steady plateau, the peak current quickly decreased as the deposition potential increased to −1.0 V. This was the result of the hydrogen evolution background that occurred at more negative potentials [24]. Thus, deposition potential at −1.2 V was considered to be the optimal accumulation potential for the determination.

#### 3.2.2. Optimization of Accumulation Time

**Figure 7.**
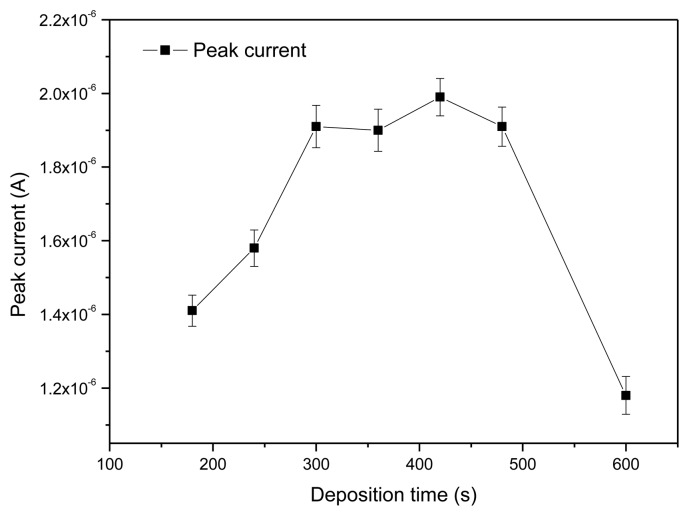
Effect of deposition time on the peak current response. The data for respective error bar were obtained from four separate experiments.

The peak current responses of the rGO-SPCE using square wave voltammetry (SWV) were measured at different deposition time. As shown in Figure 7, with increasing of deposition time from 180 s to 420 s, the stripping peak current gradually increased.

When the deposition time range was from 300 s to 480 s, the peak current response was at a plateau of high amplitude, and the peak current reached maximum when deposition time was 420 s. The peak current decreased quickly after the deposition time was above 500 s. Thus, 420 s was chosen as the optimal accumulation time.

#### 3.2.3. Detection of Pb^2+^ in Standard Solution

**Figure 8.**
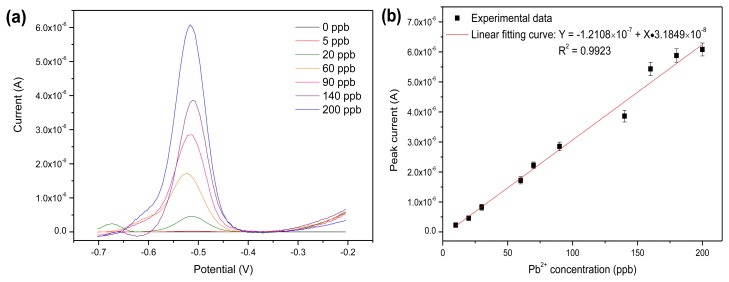
(**a**) SWV of rGO-SPCE in NaOH/HAc solution containing various concentrations of Pb^2+^; (**b**) The calibration curve of the linear dependence of cathodic peak current on Pb^2+^ concentrations. The data for respective error bar were calculated from four separate experiments.

Under the optimal operating conditions, the rGO-SPCE was applied for the detection of Pb^2+^ by using SWASV. Figure 8a shows the stripping voltammograms for different concentrations of Pb^2+^ on the rGO-SPCE. A well-defined peak at −0.50 V corresponded to the stripping peak current of Pb^2+^. Figure 8b shows the linear fitting result between the response of the rGO-SPCE and Pb^2+^ concentrations. The calibration curve (Figure 8b) showed that the linear response range of the rGO-SPCE to Pb^2+^ ranged from 5 to 200 ppb. A detection limit of 1 ppb was obtained with the calculation based on signal-noise ratio (S/N) equal to 3. Compared to the previously reported results from using different types of modified screen-printed electrodes [12,14–16], the rGO modified SPCE displayed a wider linear range for Pb^2+^ detection while maintaining a high sensitivity, as shown in Table 2.

### 3.3. Interference Test

**Figure 9.**
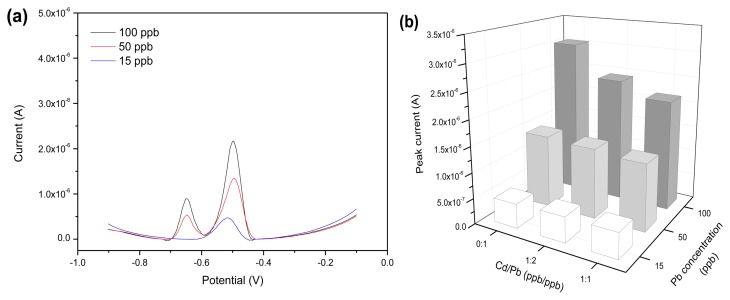
(**a**) SWV of rGO-SPCE for Pb^2+^ solution with the coexistence of the same concentration of Cd^2+^; (**b**) The analysis result of interference test data in the presence of Cd^2+^ at different concentrations.

**Figure 10.**
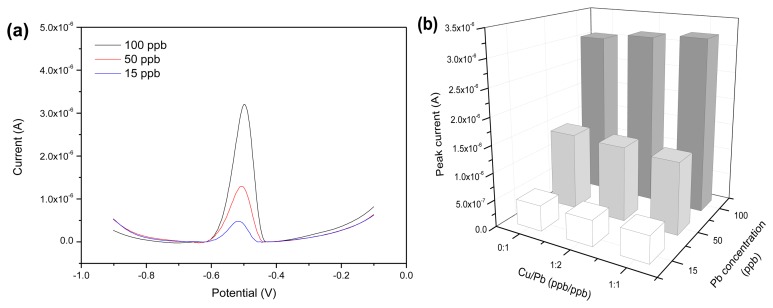
(**a**) SWV of rGO-SPCE for Pb^2+^ solution with the coexistence of the same concentration of Cu^2+^; (**b**) The analysis result of interference test data in the presence of Cu^2+^ at different concentrations.

The influence effect of the heavy metal ions Cd^2+^ and Cu^2+^ in the solution on the detection of Pb^2+^ was investigated. As shown in Figure 9a, the stripping potential of Cd^2+^ was at −0.65 V, which was staggered with respect to that of Pb^2+^. When the concentration of Cd^2+^ was no more than 50 ppb, there was no interference on the measurement results of Pb^2+^. As the concentration of Cd^2+^ increased to 100 ppb, the stripping peak current of Pb^2+^ was reduced obviously, indicating the interference effect of the presence of Cd^2+^ at high concentrations. In other words, a competitive adsorption behavior of Cd^2+^ and Pb^2+^ was found on rGO-SPCE surface when Cd^2+^ was at high concentrations. Figure 9b demonstrates the analysis result of interference test data in the presence of different Cd^2+^ concentrations. Similarly, examination of possible interferences on Pb^2+^ solutions in the presence of different Cu^2+^ concentrations was tested. Figure 10a shows that no stripping peaks were seen for Cu^2+^ for the concentration range examined. A previous report showed that the Cu^2+^ stripping peak was at −0.18 V by utilizing screen-printed carbon electrode [25]. The rGO-SPCE displayed no selectivity toward Cu^2+^ in the solution. Moreover, the Cu^2+^ at concentrations below 100 ppb had no marked effect on the Pb^2+^ stripping peak at −0.50 V, and the analysis result of interference test data is shown in Figure 10b.

### 3.4. Detection of Pb^2+^ in Real Food Samples

The pre-treated samples were detected by using the rGO modified SPCE with SWV. In order to verify the reliability of electrochemical analysis method, the results were compared with those obtained from graphite furnace atomic absorption spectrometry (GFAAS). As shown in Table 3, the experimental results showed that the response values of rGO-SPCE sensor were very close to those determined using the GFAAS method. The small value of relative standard deviation (R.S.D.) suggests the good stability of the as-prepared rGO-SPCE. The amount of Cd^2+^ in real samples, which is considered as the main interference for Pb^2+^ detection, was measured by GFAAS. It was found that the Cd^2+^ concentration was about 1 ppb in tap water, 0.5 ppb in fruit juice, 8 ppb in preserved eggs, 1 ppb in black tea, and 5 ppb in green tea. The acceptable recovery indicated the Cd^2+^ of low concentrations in the real sample had negligible effects on the electrode performance, and the as-prepared electrode shows the promising possibilities for practical applications.

## 4. Conclusions

The study found that modification of screen-printed carbon electrode by reduced graphene oxide can significantly improve the electrochemical properties of the electrode. The rGO-SPCE displayed a wide linear response range for detection of lead ions in the solution while maintaining a high sensitivity. Moreover, Cd^2+^ at concentrations below 50 ppb and Cu^2+^ at concentrations below 100 ppb both cause minimal interference with the Pb^2+^ detection. The results on the determination of Pb^2+^ in real food samples by the disposable sensor were verified by graphite furnace atomic absorption spectroscopy. The performance characteristics indicated the electrochemical analysis method holds promise for rapid, accurate, low-cost and on-site detection of lead in foods.

## Figures and Tables

**Table 1.** A summary of Raman shift and intensity obtained from various films.

**Materials on the Working Electrode of SPCE**	**D Band**		**G Band**		**I_D_/I_G_**

	**Shift/cm^−1^**	**Intensity/a.u.**	**Shift/cm^−1^**	**Intensity/a.u.**	
GO film	1,333.9	16,723.7	1,597.7	15,870.7	1.05
rGO film before Pb^2+^ detection	1,339.9	13,699.5	1,604.7	12,386.7	1.10
rGO film after Pb^2+^ detection	1,351.1	1,069.0	1,598.7	993.6	1.07

**Table 2.** The Pb^2+^ detection performance of the as-prepared sensor, compared with the literatures.

**Functional Material on SPE**	**Linear Response Range/ppb**	**Limit of Detection/ppb**	**Reference**
Bismuth/crown ether/Nafion	500∼60,000	110	[14]
Bismuth oxide	20∼300	8	[15]
Gold film	2∼16	0.5	[12]
Carbon nanotubes	2∼100	0.2	[16]
Reduced graphene oxide	5∼200	1	This work

**Table 3.** Comparison of the results of electrochemical analysis method and GFAAS.

**Real Sample**	**GFAAS**	**Electrochemical method**		

	**Found/ppb**	**Found/ppb**	**R.S.D. (*n* = 4)/%**	**Recovery/%**
Tap water	0.98	1.09	1.93	/
Tap water added 5 ppb	6.04	6.22	1.71	102.13
Tap water added 15 ppb	16.75	17.95	2.30	111.56
Tap water added 45 ppb	46.89	43.67	2.54	94.75
Orange juice	Undetected	Undetected	/	/
Orange juice added 5 ppb	5.28	5.71	1.43	114.20
Orange juice added 15 ppb	13.90	13.15	2.17	87.67
Orange juice added 45 ppb	46.24	46.85	3.55	104.11
Apple juice	Undetected	Undetected	/	/
Apple juice added 5 ppb	5.16	5.32	2.01	106.40
Apple juice added 15 ppb	15.98	15.39	2.41	102.60
Apple juice added 45 ppb	46.73	47.56	3.13	105.69
Preserved eggs (Brand A)	0.20	Undetected	/	/
Preserved eggs (Brand B)	0.40	Undetected	/	/
Preserved eggs (Brand C)	0.91	Undetected	/	/
Black tea	11.57	12.43	1.63	/
Green tea	55.32	56.10	1.98	/
